# Robot-assisted vs laparoscopic bariatric procedures in super-obese patients: clinical and economic outcomes

**DOI:** 10.1007/s11701-023-01748-y

**Published:** 2024-01-17

**Authors:** Giuseppe Marincola, Priscilla Francesca Procopio, Francesco Pennestrì, Pierpaolo Gallucci, Nikolaos Voloudakis, Luigi Ciccoritti, Francesco Greco, Giulia Salvi, Francesca Prioli, Carmela De Crea, Marco Raffaelli

**Affiliations:** 1 Centro Dipartimentale di Chirurgia Endocrina e dell’Obesità, U.O.C. Chirurgia Endocrina e Metabolica, Fondazione Policlinico Universitario Agostino Gemelli IRCCS, Università Cattolica del Sacro Cuore, L.go A. Gemelli 8, 00168 Rome, Italy; 2 Centro di Ricerca di Chirurgia delle Ghiandole Endocrine e dell’Obesità, Università Cattolica del Sacro Cuore, Rome, Italy

**Keywords:** Robot-assisted, Roux-en-Y Gastric Bypass (RYGB), Single Anastomosis Duodeno-Ileal Bypass with Sleeve Gastrectomy (SADI-S), Super-obese, Outcomes, Cost analysis

## Abstract

The increased operative time and costs represent the main limitations of robotic technology application to bariatric surgery. Robotic platforms may help the surgeon to overcome the technical difficulties in super-obese (SO, BMI ≥ 50 kg/m^2^) patients, in which multi-quadrant operations could be challenging. We aimed to evaluate the effect of robot-assisted (R) versus laparoscopic (L) approaches in Single Anastomosis Duodeno-Ileal Bypass with Sleeve Gastrectomy (SADI-S) and Roux-en-Y Gastric Bypass (RYGB) in SO and Super-Super Obese (SSO, BMI ≥ 60 kg/m^2^) patients in terms of outcomes and cost-effectiveness. Bariatric procedures performed from 2012 to 2023 were retrospectively reviewed. The inclusion criteria were BMI ≥ 50 kg/m^2^, primary SADI-S or RYGB. Operative time (OT), early complications, post-operative stay (POS), overall costs and follow-up data were analyzed. A subgroup analysis for surgical procedures and SSO patients was also performed. Among 4596 patients, 174 RYGB and 91 SADI-S in BMI ≥ 50 kg/m^2^ patients were selected. After Propensity Score Matching analysis, two groups of patients were identified (laparoscopic and robot-assisted), each one composed of 18 RYGB and 26 SADI-S. Intraoperative and post-operative complication rates and POS were comparable. Mean OT was longer in robotic procedures compared with laparoscopy (199.1 ± 65.7 and 109.5 ± 39.1 min, respectively, *p* < 0.001). The difference in OT was eliminated after only SSO patients were included in the analysis (172.7 ± 24.1 vs 152.6 ± 26.2 min for R-SADI-S and L-SADI-S, respectively, *p* = 0.107). Robotic surgeries were associated with higher costs (8134.6 ± 1886.7 and 2386.7 ± 388.2 € in R-RYGB and L-RYGB, respectively; 7996.6 ± 873.1 and 3954.6 ± 631.1 € in R-SADI-S and L-SADI-S). Despite increased costs, robotic approach may represent an added value in more complex cases such as SSO patients.

**Supplementary Information:**

The online version contains supplementary material available at 10.1007/s11701-023-01748-y.

## Introduction

Obesity is a growing concern worldwide, with its incidence increasing at an alarming rate [1, 2]. In the United States, nearly 41.9% of adults are considered obese, with severe obesity incidence doubling from 4.7 to 9.2% between 2000 and 2020 [1]. By 2030, severe obesity is predicted to be the most common type of obesity, with a 130% increase compared to 33% for obesity [2]. Moreover, super-obese (SO) patients defined by a Body Mass Index (BMI) ≥ 50 kg/m^2^ and super-super-obese (SSO) patients (BMI ≥ 60 kg/m^2^) have been registered with an even larger increase in incidence. As a consequence, the associated direct and indirect costs, including those related to metabolic complications, are creating a burden on the healthcare system [3–5].

Surgical management of SO and SSO patients often encounters significant challenges, and the efficacy of restrictive procedures remains controversial in these patients [6–8].

Despite several surgical options currently available to achieve bariatric and metabolic outcomes, to date, the most appropriate procedure for the surgical treatment is still unclear in this population [9, 10].

Indeed, Roux-en-Y Gastric Bypass (RYGB) is a standardised bariatric procedure with demonstrated effectiveness in weight loss and comorbidity resolution in these patients [11]. On the other hand, the hypo-absorptive effect of Biliopancreatic Diversion with Duodenal Switch (BPD/DS) has been shown to be more effective in achieving weight loss compared to restrictive procedures alone [12]. BPD/DS is a complex multi-quadrant surgery that involves significant anatomical changes and leads to an increased risk of post-operative surgical and metabolic complications. The Single Anastomosis Duodeno-Ileal Bypass with Sleeve Gastrectomy (SADI-S) procedure was developed as a viable alternative to the BPD/DS technique. Its primary aim was to reduce the complexity of the surgical process while still achieving comparable bariatric and metabolic outcomes. Encouraging long-term results over a period of 10 years have been seen [13]. Such surgical procedure is mostly indicated for challenging bariatric cases, including patients with a BMI of 50 kg/m^2^ or higher, metabolic patients and those requiring revisional surgery [14, 15].

Conventional laparoscopy technical limitations may be further enhanced when dealing with obese and particularly SO patients. These limitations include restricted space due to an enlarged liver, intra-abdominal fat and a thick abdominal wall. Such factors increase the difficulty of manoeuvring instruments used in minimally invasive surgery, so reconstructive times may result challenging [16, 17]. In addition, current evidences suggest that laparoscopic bariatric procedures are related to an overall complication and leaks rates of up to 20 and 5.1%, respectively, with prolonged hospital stays, reoperations and even life-threatening complications [18]. Therefore, the application of advanced technologies in bariatric surgery may help achieve optimal clinical outcomes, particularly for this specific patient’s category [19].

As a consequence, robotic systems have been gaining ground in recent years due to the enhanced dexterity and precision in tissue manipulation, thus offering advantages in difficult-to-access anatomical regions during bariatric surgery. This may result in lower conversion rates and potentially fewer short-term complications [14, 20, 21].

Unfortunately, the high costs of robotic procedures still represent a limit for their routinary use, even in challenging patients, who would benefit from the related-advantages of such technology to perform multi-quadrant surgeries [19, 26, 27].

The aim of the present study was to compare the robot-assisted versus laparoscopic approach to SADI-S and RYGB in SO and SSO patients in terms of cost-effectiveness and perioperative outcomes.

## Materials and methods

Laparoscopic RYGB (L-RYGB) was first introduced in our clinical practice in January 2012, followed by robotic RYGB (R-RYGB) in January 2013, robotic SADI-S (R-SADI-S) in July 2016, and laparoscopic SADI-S (L-SADIS-S) in February 2017.

In our Institution, data from all patients scheduled for bariatric surgery were prospectively collected in a specifically designed and de-identified database.

### Study population

A cumulative total of 4596 individuals underwent bariatric surgery at our institution between January 2012 and July 2023.

The individuals enrolled in our research study satisfied the established criteria for bariatric surgery as outlined by the consensus and national recommendations of the Italian Society of Bariatric Surgery and Metabolic Disorders (SICOb) [source: SICOb guidelines, 2016 at https://www.sicob.org/00_materiali/linee_guida_2016.pdf].

All adult patients with a BMI ≥ 50 kg/m^2^ who were scheduled for minimally invasive RYGB or SADI-S as primary surgery between January 2012 and July 2023 were candidates for inclusion.

Patients who underwent open bariatric procedures, revisional surgery, bariatric procedures different from RYGB and SADI-S and patients who underwent concomitant procedures at the time of bariatric surgery were excluded from the analysis.

In this series, all robotic procedures (RYGB and SADI-S) and all L-SADI-S have been performed by the same senior minimally invasive expert surgeon (M.R.).

In our Centre, all L-RYGBs on SO and SSO patients were performed by surgeons who completed a preliminary learning curve of at least 100 L-RYGBs on patients with a BMI < 50 kg/m^2^.

In details, among 4016 primary minimally invasive bariatric procedures, 174 and 91 patients who underwent RYGB and SADI-S, respectively (intention to treatment analysis) met the inclusion/exclusion criteria. More specifically, among 265 SO patients, 14 patients had a BMI > 60 kg/m2 (SSO).

Patients were classified into two separate cohorts according to the surgical approach: the robot-assisted group and the laparoscopic group.

Preoperative characteristics included gender, age and BMI, comorbidities such as hypertension, OSAS and type 2 diabetes mellitus (T2DM) and previous abdominal surgery. To determine the effectiveness of surgery, we thoroughly assessed various intraoperative factors, such as the operative technique and surgical approach, as well as the duration of the operation (OT) and any potential complications encountered during the procedure. Post-operative parameters included post-operative intensive care unit (ICU) stay, post-operative hospital stay (POS), early (within 30 days) minor and major complications and 12 months-follow-up data.

Furthermore, the study included a cost analysis comparing the use of robot-assisted versus laparoscopic techniques for each procedure.

To overcome possible selection biases on outcomes, the robotic and laparoscopic patients were matched for gender, age, BMI, comorbidity and surgical procedure with propensity score matching (PSM) analysis.

Follow-up was performed through outpatient consultations with a multidisciplinary team 30 days, 3 months, 6 months and 12 months after surgery to monitor patients’ weight loss, metabolic disorders, comorbidities, therapy, diet compliance and complications.

For this study, the follow-up was closed on 31st August 2023.

The study was done in compliance with the ethical principles outlined in the Declaration of Helsinki and obtained approval from our institution’s ethical committee based on study protocols 00013532/23 and 00108953/23. All individual participants included in this article provided informed consent.

### Study end-points

We aimed to evaluate the complications rate between the laparoscopic and robot-assisted approaches as our primary end-point. Our secondary end-point was to compare the two approaches in terms of OT, hospital stay and costs.

### Definitions

The OT is defined as the interval from the initial incision to the final closure of the surgical site (skin to skin), encompassing the docking step for robotic procedures. Post-operative complications (occurring until the 30th post-operative day) were recorded according to the Clavien–Dindo classification [22] and considered minor (Clavien–Dindo grade I–II) and major (Clavien-Dindo grade III-IV) complications.

To calculate the percentage of Excess Weight Loss (%EWL), we used the following formula: (starting weight −current weight)/(starting weight −weight for a BMI of 25 kg/m^2^) × 100. The weight at a BMI of 25 kg/m^2^ is considered the ideal body weight.

The economic model to perform the cost analysis was previously described in extenso [27]. Our administrative service assessed the costs for each patient using a combination of micro-costing and gross costing. In Italy, reimbursement for bariatric surgery is a flat rate of 5681.3 €, regardless of the procedure used or the complications. Our hospital pays a fixed salary to the operating room staff, and the cost analysis included anaesthesia, surgery, and scrub nurse costs. Hospital stay costs were evaluated using a combination of micro-costing and gross costing for drugs, exams and professional and accommodation costs.

### Surgical techniques

Before the procedure, informed consent was acquired from all patients. The specific surgical approach (laparoscopic vs. robot-assisted) and procedure (RYGB vs. SADI-S) was chosen based on the patient’s characteristics and the surgeon’s and patient’s preference. All the robotic procedures included in the present study were performed using Da Vinci Intuitive^®^ platform. More in detail, the da Vinci Si platform was used until 2014, when the da Vinci Xi platform replaced it.

The description of the surgical technique of R-SADI-S and L-SADI-S has already been reported [14, 15], as well as L-RYGB [23, 24].

In our routine clinical practice, R-RYGB is realized with a double-loop technique. The gastric pouch is created by means of a linear stapler with three 60 mm cartridges, with the first one being horizontal and the other two being vertical. A 40 Fr orogastric bougie is used for the calibration. A double-layer, hand-sewn termino-lateral antecolic gastro-jejunal anastomosis is performed between the gastric pouch and a jejunal loop located 75–120 cm distal to the Treitz ligament, using a 3/0-barbed suture. The side-to-side jejuno-jejunal anastomosis is created 150 cm from the previous one using a 60 mm linear stapler. The defects are closed with a barbed running suture. The anastomosis is verified for integrity using blue methylene and a pneumatic test.

We have adopted laparoscopic staplers, regardless of whether the procedure was laparoscopic or robot-assisted. For R-SADI-S, the gastrocolic ligament's dissection and the greater curvature's preparation are carried out using Da Vinci Vessel Sealer. Instead, for R-RYGB, the dissection for the preparation of the gastric pouch is achieved using Da Vinci Fenestrated bipolar forceps.

The post-operative protocol has been elucidated in extenso in prior scientific studies [19, 20, 25, 30, 31]. All RYGB patients received FitForMe WLS Forte^®^ as vitamins and minerals supplementation customized for this bariatric procedure (see https://fitforme.it/product/wls-forte/?_gl=1*12cjjqc*_up*MQ..&gclid=CjwKCAjw1t2pBhAFEiwA_-A-NHqOIVB1CPEiVIzNVJysVEaQrxrCjX_OPjpszcp0CfFu7NwCs0mZlBoCJ20QAvD_BwE#product-tabs for details of composition). For optimal results, it is advised to consume one capsule of FitForMe WLS Forte^®^ on a daily basis. This recommended dosage ensures that the supplement is taken in the correct quantity.

### Statistical analysis

PSM was conducted with the 1:1 nearest-neighbour matching approach, with a calliper of 0.01 and discarding observations from both groups. The treatment variable in the regression model of PSM was the surgical approach, specifically comparing robot-assisted surgery to laparoscopic surgery. The regression model incorporated confounders, namely gender (male vs. female), BMI, surgical method (RYGB versus SADI-S) and comorbidities, because of their potential impact on the end-points under investigation.

A bivariate analysis was performed to assess and evaluate the preoperative features, operating factors and post-operative variables. The Shapiro–Wilk test was employed to assess the conformity of the data to a normal distribution. The comparison of categorical variables was conducted using Fisher’s exact test and Chi-square test, whereas continuous data were presented as mean (± standard deviation, SD) or median (interquartile range, IQR). To assess the differences between continuous variables, the statistical methods employed were the paired sample *t *test or the Mann–Whitney *U* test, chosen based on the data distribution characteristics of the population under investigation.

We performed a subgroup analysis according to the type of surgical procedure (RYGB and SADI-S) and BMI (SO and SSO patients).

To adhere to the established economic report methodology, we reported means ± standard deviation (95% confidence interval) for non-parametric variables in the cost analysis. Patient charts and electronic databases were used to collect demographic and clinical data. Statistical analysis and PSM were performed using Stata version 17.0 (StataCorp, College Station, Texas, 77845, USA).

The statistical analyses conducted in this study were two-tailed and the threshold for determining statistical significance was set at *p *≤ 0.05.

## Results

**Fig. 1:**
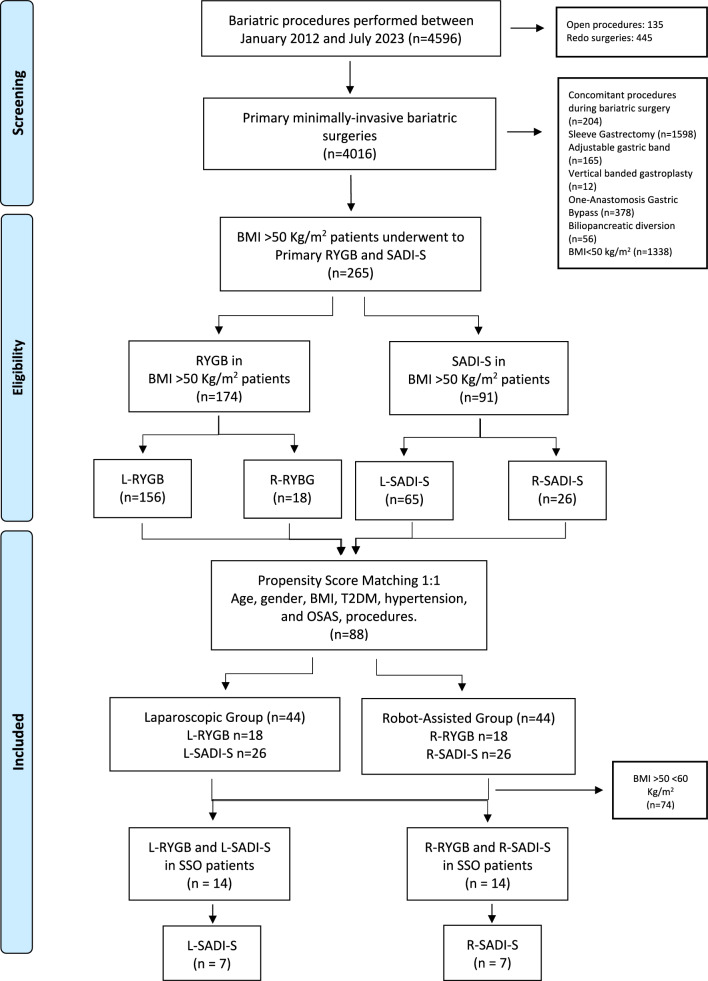
Study patient’s flowchart diagram

From January 2012 to July 2023, 4596 bariatric procedures were performed. A total of 265 BMI ≥ 50 kg/m^2^ patients were selected according to inclusion criteria. Laparoscopic procedures were performed in 221 (83.4%) patients, while robot-assisted procedures in 44 (16.6%) patients. The laparoscopic group included 156 (70.6%) RYGB and 65 (29.5%) SADI-S, while the robot-assisted group included 18 (40.9%) RYGB and 26 (59.1%) SADI-S. After PSM, the study population consisted of 88 patients: 44 in the laparoscopic group and 44 in the robot-assisted group. Each subgroup included 18 RYGB and 26 SADI-S. Figure 1 reports the study patient’s flowchart diagram. Table 1 shows the characteristics of the study’s population. There were 49 (55.7%) females and 39 (44.3%) males. The mean age was 44.5 ± 9.6 years and the mean BMI was 55.6 ± 4.8 kg/m^2^. Overall, the mean OT was 154.3 ± 45.1 min. There were no documented occurrences of conversions reported, either from laparoscopic to open surgery or from robotic to laparoscopic/open surgery. The median POS was 3 (2–4) days. No readmissions after discharge were registered. A total of 5 patients, accounting for 5.7% of the sample, experienced complications following surgery. The observed death rate within a 30-day period was found to be nil.

**Table 1:** Clinicopathological characteristics and post-operative outcome between Laparoscopic Group and Robot-assisted Group in super-obese patients after propensity matching score analysis

	Total	Laparoscopic group	Robot-assisted group	*p* value*
Number of patients	88	44	44	
Mean age (± SD), years	44.5 ± 9.6	44.2 ± 9.3	44.7 ± 10.1	0.809
Male/female	49 (55.7%)/39 (44.3%)	24 (54.5%)/20 (45.5%)	25 (56.8%)/19 (43.2%)	0.831
Mean BMI (± SD), kg/m^2^	55.6 ± 4.8	56.1 ± 5.2	55.1 ± 4.7	0.346
SO (BMI 50–59.9 kg/m^2^)	74 (84.1%)	37 (84.1%)	37 (84.1%)	
SSO (BMI ≥ 60 kg/m^2^)	14 (15.9%)	7 (15.9%)	7 (15.9%)	1
Preoperative comorbidity (yes/no)	56 (63.6%)/32 (36.4%)	28 (63.6%)/16 (36.4%)	28 (63.6%)/16 (36.4%)	1
OSAS (yes/no)	40 (45.5%)/48 (54.5%)	19 (43.2%)/25 (56.8%)	21 (44.7%)/23 (52.3%)	0.669
Hypertension (yes/no)	39 (44.3%)/49 (55.7%)	19 (43.2%)/25 (56.8%)	20 (45.5%)/24 (54.5%)	0.831
Type 2 diabetes mellitus (yes/no)	28 (31.2%)/60 (68.2%)	12 (27.3%)/32 (72.7%)	16 (36.4%)/28 (63.6%)	0.362
Previous abdominal surgery (yes/no)	31 (35.3%)/57 (64.7%)	15 (34.1%)/29 (65.9%)	16 (36.4%)/28 (63.6%)	0.824
Procedure:				
RYGB	36 (40.9%)	18 (40.9%)	18 (40.9%)	1
SADI-S	52 (59.1%)	26 (59.1%)	26 (59.1%)	
Mean operative time (± SD) min	154.3 ± 45.1	109.5 ± 39.1	199.1 ± 65.7	< 0.001
Intraoperative complications (yes/no)	0	0	0	1
Post-operative total complications (yes/no)	5 (5.7%)/83 (94.3%)	1 (2.3%)/43 (97.7%)	4 (9.1%)/40 (90.9%)	0.359
Post-operative minor complications (yes/no)	2 (2.3%)/86 (97.7%)	0	2 (4.5%)/42 (95.5%)	0.493
Post-operative major complications (yes/no)	3 (3.4%)/85 (96.6%)	1 (2.3%)/43 (97.7%)	2 (4.5%)/42 (95.5%)	0.999
Post-operative ICU (yes/no)	4 (4.5%)/84 (95.5%)	0	4 (9.1%)/40 (90.9%)	0.116
Median post-operative hospital stay (IQR) days	3 (2–4)	3 (3–4)	3 (2–4)	0.469
Readmissions (yes/no)	0	0	0	1
Mean overall cost (± SD), euros	5692.8 ± 1123.3	3313.1 ± 911.8	8052.6 ± 1234.7	< 0.001

*SD* Standard deviation, *IQR* 75% interquartile range, *BMI* Body mass index, *SO* Super-obese, *SSO* Super super-obese, *OSAS* Obstructive sleep apnea syndrome, *ICU* Intensive care unit, *RYGB* Roux-en-Y gastric bypass, *SADI-S* Single anastomosis duodeno-ileal bypass with sleeve gastrectomy, *p values refer to comparison between Laparoscopic group and Robotic group

In Table 1, the comparative analysis between laparoscopic and robot-assisted groups is also reported. The two groups were comparable in terms of age, gender distribution, BMI and preoperative comorbidities (*p* = 0.809, 0.831, 0.346, 1, respectively). SSO patients only underwent SADI-S procedure (7 L-SADI-S and 7 R-SADI-S). In both groups, no intraoperative complications were registered. The mean OT was longer in the robot-assisted group: 199.1 ± 65.7 min vs 109.5 ± 39.1 min in the laparoscopic group (*p *< 0.001). After subgroup analysis for surgical procedures (RYGB and SADI-S), higher OT was registered for SADI-S (Tables 2 and 3). However, in the subgroup analysis considering BMI ≥ 60 kg/m^2^, the OT was similar between the two approaches (Table 4). There was no evidence of significant differences between the two groups in terms of post-operative complications. Two (2.3%) minor complications were registered in the robot-assisted group (grade II). Major complications occurred in one (2.3%) laparoscopic group patient (grade IV) and in 2 (4.5%) robotic group patients (grade IIIa and IV). POS was comparable among the two groups (3 days vs. 3 days, *p* = 0.469). A comprehensive account of the post-operative complications can be found in the supplementary materials.

**Table 2:** Clinicopathological characteristics and post-operative outcome between Laparoscopic and Robot-assisted RYGB subgroups

	L-RYGB group	R-RYGB group	*p* value
Number of patients	18	18	
Mean age (± SD), years	43.5 ± 9.1	42.1 ± 11.1	0.681
Male/female	7 (38.9%)/11 (61.1%)	9 (50%)/9 (50%)	0.508
Mean BMI (± SD), kg/m^2^	55.3 ± 4.2	54.5 ± 3.2	0.534
SO (BMI 50–59.9 kg/m^2^) SSO (BMI ≥ 60 kg/m^2^)	18 (100%) 0	18 (100%) 0	1
Preoperative comorbidity (yes/no)	15 (83.3%)/3 (16.7%)	14 (77.8%)/4 (22.2%)	0.737
OSAS (yes/no)	12 (66.7%)/6 (33.3%)	13 (72.2%)/5 (27.8%)	0.721
Hypertension (yes/no)	9 (50%)/9 (50%)	10 (55.5%)/8 (44.5%)	0.742
Type 2 diabetes mellitus (yes/no)	6 (33.3%)/12 (66.7%)	8 (44.4%)/10 (55.6%)	0.499
Previous abdominal surgery (yes/no)	6 (33.3%)/12 (66.7%)	4 (22.2%)/14 (77.8%)	0.463
Mean operative time (± SD), min	72.8 ± 19.2	189.7 ± 34.2	< 0.001
Intraoperative complications (yes/no)	0	0	1
Post-operative total complications (yes/no)	1(5.6%)/17 (94.4%)	2 (11.1%)/16 (88.9%)	0.999
Post-operative minor complications (yes/no)	0	1/17 (%)	0.999
Post-operative major complications (yes/no)	1 (5.6%)/17 (94.4%)	1 (5.6%)/17 (94.4%)	1
Post-operative ICU (yes/no)	0	2 (11.1%)/16 (88.9%)	0.485
Post-operative hospital stay (IQR), days	3 (3–4)	3 (3–5)	0.953
Readmissions (yes/no)	0	0	1
Mean Overall cost (± SD), euros	2386.7 ± 388.2	8134.6 ± 1886.7	< 0.001

*SD* Standard Deviation, *IQR* 75% interquartile range, *BMI* Body mass index, *SO* Super-obese, *SSO* Super-super-obese, *OSAS* Obstructive sleep apnea syndrome, *ICU* Intensive care unit, *L* laparoscopic, *R* Robot-assisted, *RYGB* Roux-en-Y gastric bypass

**Table 3:** Clinicopathological characteristics and post-operative outcome between Laparoscopic and Robot-assisted SADI-S subgroups

	L-SADI-S group	R-SADI-S group	*p* value
Number of patients	26	26	
Mean age (± SD), years	44.7 ± 9.2	46.6 ± 9.4	0.464
Male/female	17 (65.4%)/9 (34.6%)	16 (61.5%)/10 (38.5%)	1
Mean BMI (± SD), kg/m^2^	56.7 ± 6.1	55.6 ± 5.7	0.504
SO (BMI 50–59.9 kg/m^2^) SSO (BMI ≥ 60 kg/m^2^)	19 (73.1%) 7 (26.9%)	19 (73.1%) 7 (26.9%)	1
Preoperative comorbidity (yes/no)	13 (50%)/13 (50%)	14 (53.8%)/12 (46.2%)	0.783
OSAS (yes/no)	7 (26.9%)/19 (73.1%)	8 (30.8%)/18 (69.3%)	0.761
Hypertension (yes/no)	10 (38.5%)/16 (61.5%)	10 (38.5%)/16 (61.5%)	1
Type 2 diabetes mellitus (yes/no)	6 (23.1%)/20 (76.9%)	8 (30.8%)/18 (69.2%)	0.535
Previous abdominal surgery (yes/no)	9 (34.6%)/17 (65.4%)	12 (46.2%)/14 (53.8%)	0.401
Mean operative time (± SD), min	135.5 ± 31.5	205.7 ± 60.1	< 0.001
Intraoperative complications (yes/no)	0	0	1
Post-operative total complications (yes/no)	0	2 (7.7%)/24 (92.3%)	0.489
Post-operative minor complications (yes/no)	0	1 (3.8%)/25 (96.2%)	0.999
Post-operative major complications (yes/no)	0	1 (3.8%)/25 (96.2%)	0.999
Post-operative ICU (yes/no)	0	2 (7.7%)/24 (92.3%)	0.489
Median post-operative hospital stay (IQR), days	2 (2–4)	2 (2–3)	0.425
Readmissions (yes/no)	0	0	1
Mean overall cost (± SD), euros	3954.6 ± 631.1	7996.6 ± 873.1	< 0.001

*SD* Standard deviation, *IQR* 75% interquartile range, *BMI* Body mass index, *SO* Super-obese, *SSO* Super-super-obese, *OSAS* Obstructive sleep apnea syndrome, *ICU* Intensive care unit, *L* laparoscopic, *R* Robot-assisted, *SADI-S* Single anastomosis Duodeno-Ileal Bypass with Sleeve Gastrectomy

**Table 4:** Clinicopathological characteristics and post-operative outcome between Laparoscopic and Robot-assisted SADI-S subgroups in super-super-obese patients

	SSO L-SADI-S group	SSO R-SADI-S group	*p* value
Number of patients	7	7	
Mean age (± SD), years	37.5 ± 8.7	45.28 ± 8.2	0.072
Male/female	4 (57.1%)/3 (42.9%)	3 (42.9%)/4 (57.1%)	0.606
Mean BMI (± SD), kg/m^2^	64.6 ± 3.1	62.9 ± 2.9	0.267
Preoperative comorbidity (yes/no)	6 (85.7%)/1 (14.3%)	5 (71.4%)/2 (28.6%)	0.393
OSAS (yes/no)	6 (85.7%)/1 (14.3%)	5 (71.4%)/2 (28.6%)	0.393
Hypertension (yes/no)	4 (57.1%)/3 (42.8%)	4 (57.1%)/3 (42.8%)	1
Type 2 diabetes mellitus (yes/no)	4 (57.1%)/3 (42.8%)	5 (71.4%)/2 (28.6%)	0.591
Previous abdominal surgery (yes/no)	3 (42.8%)/4 (57.1%)	3 (42.8%)/4 (57.1%)	1
Mean operative time (± SD), min	152.6 ± 26.2	172.7 ± 24.1	0.107
Intraoperative complications (yes/no)	0	0	1
Post-operative total complications (yes/no)	0	0	1
Post-operative minor complications (yes/no)	0	0	1
Post-operative major complications (yes/no)	0	0	1
Post-operative ICU (yes/no)	1 (14.3%)/6 (85.7%)	0	0.317
Median post-operative hospital stay (IQR), days	3 (2–4)	2 (2–4)	0.374
Readmissions (yes/no)	0	0	1
Mean overall cost (± SD), euros	3710.6 ± 770.3	7632.9 ± 1412.8	< 0.001

*SD* Standard deviation, *IQR* 75% interquartile range, *BMI* Body mass index; *SO* Super-obese, *SSO* Super super-obese, *OSAS* Obstructive sleep apnea syndrome, *ICU* Intensive care unit, *L* laparoscopic, *R* Robot-assisted, Single Anastomosis Duodeno-Ileal Bypass with Sleeve Gastrectomy

The follow-up time of the entire series was concluded in 31st August 2023, without lost-at-follow-up patients. Further follow-up data in terms of bariatric outcomes are reported in the supplementary materials in details, including only patients with 12 months-follow-up.

Finally, the cost analysis showed a statistically significant difference, with a mean overall cost of 3313.1 ± 911.8 € for the laparoscopic procedure and of 8052.6 ± 1234.7 € for the robot-assisted group. Further differences in terms of overall costs among the subgroups are summarised in Tables 2, 3 and 4. Table 5 summarises a comparative analysis related to the specific item cost between Laparoscopic Group and Robot-Assisted Group.

**Table 5:** Comparative analysis related to the specific item cost between Laparoscopic Group and Robot-assisted Group

	Laparoscopic group	Robot-assisted group
Mean hospital stay cost (€)	998	912
Mean operating room occupation cost (€)	1012	2103
Mean medical procedure cost (€)	211	287
Mean intensive care unit cost (€)	–	285
Mean medical devices’ cost (€)	1034	4390

## Discussion

This retrospective cohort study presents a comparative analysis of robot-assisted and laparoscopic bariatric surgeries conducted in a high-volume center between January 2012 and July 2023. The overall bariatric procedures of our Institution during the study period were 4596, with a surgical annual volume which progressively increased from 200 to about 700 cases.

Concerning the primary outcome of the study, we found comparable post-operative complication rates: four (9.1%) patients vs. one (2.3%) patient for robot-assisted and laparoscopic groups, respectively. Post-operative complications rates were also comparable among the subgroups, occurring in one (5.6%) patient and two (11.1%) patients of the L-RYGB and R-RYGB group, respectively, in the RYGB group and in two (7.7%) R-SADI-S group patients among the SADI-S population.

Major post-operative complications were observed in one (5.6%) L-RYGB patient in one (5.6%) R-RYGB patient among the RYGB group, and in one (3.8%) R-SADI-S patient.

Our experience confirms the reports of the majority of studies on topic stating that robotic approach to bariatric surgery is a safe procedure with acceptable perioperative complications.

Most of the previous reports showed similar results in terms of post-operative complications between the robotic and the laparoscopic procedures [26–28]. Nelson et al. [29] analyzed 69 SO patients who underwent laparoscopic and robot-assisted SADI-S and demonstrated that both surgical approaches are feasible and safe options for these patients, with a mean post-operative hospital stay of 4.3 ± 2.6 days, 30 days-readmission rate of 4.3% and the 30 days-reoperation rate of 5.8%. Ayloo et al. [30] also compared the outcomes of 90 R-RYGB and 45 L-RYGB, with similar results to our experience in terms of early morbidity. A meta-analysis of Bertoni et al. [31] considered a total of 29,890 patients, including 2459 and 27,431 robotic and laparoscopic bariatric surgery, with similar baseline characteristics. The rate of early post-operative complications was analyzed, showing no significant difference between the two groups (8.1 vs. 7.5%), neither in terms of conversion to open surgery, as well (0.5 vs. 0.3%).

Our analysis also confirms that robot-assisted surgery is related to longer OT, with values of 109.5 ± 39.1 min and 199.1 ± 65.7 min in laparoscopic and robot-assisted procedures, respectively. More specifically, among the RYGB subgroup, we registered OT of 72.8 ± 19.2 min in L-RYGB patients and 189.7 ± 34.2 min in R-RYGB patients (*p *< 0.001), while among the SADI-S group 133.5 ± 31.5 min in L-SADI-S patients and 205.7 ± 60.1 min in R-SADI-S patients (*p *< 0.001).

It is acknowledged that the OT for robotic surgery is longer compared to laparoscopy, especially at the initial phases of application [31]. Delving deeper, several studies underlined that the docking step is related to a significant increase of OT [32, 33]. In our previous experience, we underlined that despite robotic technology-associated longer OT, surgical complications are comparable to laparoscopy [14].

We also showed that, when case series on a long-time period are considered, differences among laparoscopic and robotic procedures in terms of hospital stay may be explained by variations of clinical protocol (e.g., ERAS fast track protocol), distance between hospital and patient’s home and cultural behavior [14].

Our results are in line with other author’s reports. In Wesley Vosburg et al.’s [34] retrospective case–control study, the outcomes of 201,516 L-RYGB and 21,462 R-RYGB were compared, showing longer OT in the robotic group when compared to the laparoscopic one, with an average OT increase of 33% (40.5 minutes) (*p* = 0.001) for R-RYGB. In a meta-analysis by Economopoulos et al. [40], the OT for R-RYGB and L-RYGB revealed no significant differences between the two groups. However, it was observed that the stapled anastomosis L-RYGB group had a significantly shorter OT compared to the hand-sewn anastomosis R-RYGB group (*p* = 0.001). Robotic approach to SADI-S has been described by several authors [12, 35–37], reporting similar OTs compared to our analysis, as well.

On the other hand, we reported comparable OT between the robotic and laparoscopic groups among SSO patients. Such data are probably due to the minor impact of the patient’s complexity on the robotic approach when compared to laparoscopy. Indeed, robotic procedures allow to overcome SO and SSO patients-related surgical difficulties, due to the aforementioned advantages of robotic technology [28, 38].

Furthermore, robotic surgery can provide greater comfort to surgeons compared to the traditional laparoscopic procedures, allowing for enhanced precision and control of surgical instruments. In addition, the use of 3D and high-definition visualization can provide a clearer view of the anatomy and help to identify potential complications [39].

Laparoscopic hand-sewn anastomosis, such as those performed during RYGB and SADI-S, may be physically demanding in multi-quadrant, complex operations, and lead the surgeon to perform small, precise movements for an extended period [40]. Robotic systems can reduce the physical strain, allowing the surgeon to operate from a console, using hand and foot controls. As a result, surgeon suffers from the technical challenges due to complex patients in reduced measure when compared to laparoscopic surgery, even in case of OT lengthening, thus making OT comparable among the two approaches. However, the less frequent application of robotic approach to bariatric surgery when compared to laparoscopic procedure is noteworthy, as robotic technology has been applied in less than 3% of patients in our clinical practice.

The increased costs of robotic platforms in bariatric surgery remain one of the main hurdles for widespread application. Indeed, robotic surgery can be more expensive compared to laparoscopic equipment, though the procedure-related costs can widely vary on the basis of several further factors, such as geographic location, hospital fees and insurance coverage [41, 42].

In line with the literature evidence, the analysis of our experience showed that robotic procedures presented a lesser cost margin compared to laparoscopic surgery, mainly due to the different medical devices costs [27].

Delving deeper, the laparoscopic approach-related positive marginality has been estimated for 2368.2 ± 911.8 €, while the robot-assisted procedure is related to a negative marginality of 2471.3 ± 1234.7 €.

On the other hand, we believe that the cost of robotics is justified from the inherent challenges in this particular patient’s population, providing advantages which include reduced surgeon fatigue, enhanced visualization of the operating field and improved identification of anatomical structures during surgery. Moreover, in an integrated and economically sustainable model, the positive marginality excess derived from the laparoscopic surgery can be used to sustain the robotic platforms application to complex cases. Indeed, several authors agree with our opinion by arguing that, despite the associated higher costs, robotic surgery may provide substantial benefits for challenging patients’ cohorts, such as SO and SSO patients [28].

Furthermore, it is to underline that robotic-related high costs are mainly due to a unique device dominance in clinical practice [43, 44]. The introduction of different robotic platforms, such as HUGO^™^ RAS [45] which also found their application in our personal experience (in press data), may lead to a considerable economic reduction of robotic technology in the future due to company’s competitiveness. As a result, a more sustainable application of robotic platforms to bariatric surgery and therefore their major diffusion worldwide may realize.

We believe that the present study is valuable for being a case–control, comparative study for robotic and laparoscopic bariatric surgery in a high-volume center, with a large collection of clinical data on minimally invasive procedures. The primary cause of bias in multicentric research may be related to the lack of uniformity in selection criteria, clinical management and expertise. One of the strengths of our research lies in the homogeneity of the supplied data.

It is important to highlight the limitations of this study. This retrospective analysis encompasses patients who underwent surgery over an extended period. A propensity score analysis was performed to match cases appropriately. Secondly, the limited study population might not accurately reflect the broader population, affecting the validity and reliability of our findings. Moreover, the definition of the correct sample size is critical, as it has been reported that more than 2000 patients would be necessary to observe a significant difference through a power analysis in terms of operative complications between the two approaches. This may limit the reliability of our findings.

In addition, our study may be subject to confounding biases, as further factors may explain the associations observed between our variables of interest. We attempted to control potential confounding variables through statistical analysis. Finally, it is to underline that the reported robotic procedures also include the first experiences of robotic platform at the beginning of learning curve in our clinical practice, in contrast with the more advanced expertise of laparoscopy. Such factor may be considered a further bias for the evaluation of the reported clinical outcome.

In conclusion, robotic and laparoscopic approaches to bariatric surgery are comparable in terms of post-operative complications in SO and SSO patients. Nevertheless, despite the higher costs, robotic surgery may add a noteworthy value for the treatment of challenging patients, especially in an economically sustainable model. Although we believe that our results may be promising, larger studies with wider sample size and longer follow-up are necessary to draw definitive conclusions.

## Supplementary Information

Below is the link to the electronic supplementary material.

Supplementary file1 (DOCX 181 KB)

## Data Availability

The raw data supporting the conclusion of this article will be made available by the authors, without undue reservation.
