# Supplementary material for: Robot-assisted vs laparoscopic bariatric procedures in super-obese patients: clinical and economic outcomes
Source: J Robot Surg. 2024 Jan 17;18(1):34. doi: 10.1007/s11701-023-01748-y (PMC10794378; doi:10.1007/s11701-023-01748-y)
Supplement: Supplementary file 1 — Supplementary file1 (DOCX 181 KB) [file 11701_2023_1748_MOESM1_ESM.docx]

**Comprehensive overview of complicated patients undergoing RYGB procedures:**

A 53-years-old female patient (BMI 57.5 kg/m^2^) underwent R-RYGB. The upper gastro-intestinal contrast study revealed a micro-leak, which was confirmed by abdominal CT with oral contrast. Empirical intravenous antibiotic and antifungal therapy was administrated and a nasojejunal tube was endoscopically placed until complete clinical remission. Resolution of the complication (Clavien-Dindo grade IIIa) was also confirmed through radiological control on 11^th^ POD. No further complications were observed after oral feeding was re-established nor at 12-months-follow-up.

A 34-years-old male patient (BMI 52.1 kg/m^2^) underwent R-RYGB. On 1^st^ POD he presented dyspnoea and mild desaturation. Respiratory alkalosis was reported through blood gas analysis. A chest CT scan was performed, showing sign of pulmonary embolism. The patient was conservatively treated (Clavien-Dindo grade II). No ICU care was necessary. The patient was discharged in 7^th^ POD. No further complications were observed at the 12-months-follow-up.

A 46-years-old female patient (BMI 53.3 kg/m^2^) underwent L-RYGB. On 3^rd^ POD, multiple emetic episodes and pain in supra-umbilical regional, with no gastrointestinal peristalsis at abdominal auscultation. An abdominal contrast CT scan showed the presence of incisional hernia at the trocar site with vascular impairment. An urgent exploratory laparoscopy and surgical resection was performed to treat the complication (Clavien-Dindo grade IV). Post-operative ICU care was necessary (1 day). The patient was discharged in 8^th^ POD, with no further complications at the 12 months-follow-up.

**Comprehensive overview of complicated patients undergoing SADI-S procedures:**

A 43-years-old patient (BMI 54.6 kg/m^2^) underwent R-SADI-S. On 2^nd^ postoperative day, the patient presented with repeated episodes of bile vomiting associated with pain localized at the peri-umbilical trocar site. An abdominal CT scan, showing an incarcerated incisional hernia at the trocar site. An exploratory laparoscopy and the hernia reduction was performed (Clavien-Dindo grade IV). Postoperative ICU care was necessary (1 day). She was discharged in 7^th^ POD. At the 12-months-follow-up no further complications were reported.

A 62-years-old woman (BMI 51.4 kg/m2) underwent R-SADI-S. On 2^nd^ POD the patient presented with fever, without abdominal pain at clinical evaluation. Blood tests showed elevated acute phase reactants of inflammation. Despite negative upper gastrointestinal contrast study, the persistent fever led us to opt for an abdominal and chest CT with intravenous and oral contrast, which showed pneumonia (Clavien-Dindo grade II). Intravenous antibiotic therapy was administrated, with consequent improvement of clinical conditions. The patient was discharged in 9^th^ POD. No further complications occurred at the 12-months-follow-up.

***Anthropometric results and partial or total resolution of comorbidities after 12 months follow-up of patients enrolled in our study***

Anthropometric results and partial or total resolution of comorbidities after 12 months follow-up between Laparoscopic Group and Robotic Group in Super Obese Patients after propensity matching score.

|  | Total | Laparoscopic group | Robotic group | p-value* |
| --- | --- | --- | --- | --- |
| Number of patients | 80 | 40 | 40 |  |
| BMI (± SD), kg/m^2^ | 31.8±5.8 | 32.1±5.8 | 31.8±5.9 | 0.819 |
| %EWL (± SD) | 74.3±19.1 | 72.4±20.6 | 77.1±17.1 | 0.271 |
| SD: Standard Deviation; BMI: Body Mass Index; %EWL: Percentage of Excess Weight Loss | | | | |

Anthropometric results and partial or total resolution of comorbidities after 12 months follow-up between Laparoscopic RYGB Group and Robotic RYGB Group in Super Obese patients after propensity matching score.

|  | L-RYGB group | R-RYGB group | p-value* | |
| --- | --- | --- | --- | --- |
| Number of patients | 16 | 16 |  | |
| BMI (± SD), kg/m^2^ | 32.6±6.1 | 32.2±6.5 | 0.911 | |
| %EWL (± SD) | 73.9±18.1 | 71.8±23.1 | 0.767 | |
| SD: Standard Deviation; BMI: Body Mass Index; %EWL: Percentage of Excess Weight Loss; RYGB: Roux-en-Y Gastric Bypass | | | |  |

Anthropometric results and partial or total resolution of comorbidities after 12 months follow-up between Laparoscopic SADI-S Group and Robotic SADI-S Group in Super Obese patients after propensity matching score.

|  | L-SADI-S Group | R- SADI-S group | p-value | |
| --- | --- | --- | --- | --- |
| Number of patients | 24 | 24 |  | |
| BMI (± SD), kg/m^2^ | 30.8±4.2 | 31.6±5.5 | 0.575 | |
| %EWL (± SD) | 76.8±18.6 | 74.6±19.6 | 0.693 | |
| SD: Standard Deviation; BMI: Body Mass Index; %EWL: Percentage of Excess Weight Loss; SADI-S: Single Anastomosis Duodeno–Ileal Bypass with Sleeve Gastrectomy | | | |  |

Anthropometric results and partial or total resolution of comorbidities after 12 months follow-up between Laparoscopic SADI-S Group and Robotic SADI-S Group in Super Super Obese patients after propensity matching score.

|  | SSO L-SADI-S group | SSO R-SADI-S group | p-value | |
| --- | --- | --- | --- | --- |
| Number of patients | 7 | 7 |  | |
| BMI (± SD), kg/m^2^ | 31.09±5.32 | 33.32±10.18 | 0.556 | |
| %EWL (± SD) | 79.46 ± 11.51 | 78.81 ± 18.62 | 0.928 | |
| Comorbidities Resolution (yes/no) |  |  |  | |
| Complete (yes/no) | 2 (28.6%)/ 5 (71.4%) | 1 (14.3%)/ 6 (85.7%) | 0.529 | |
| Partial (yes/no) | 4 (57.1%)/ 3 (42.9%) | 2 (28.6%)/ 5 (71.4%) | 0.298 | |
| SD: Standard Deviation; BMI: Body Mass Index; %EWL: Percentage of Excess Weight Loss; SADI-S: Single Anastomosis Duodeno–Ileal Bypass with Sleeve Gastrectomy | | | |  |
